# Cognitive Impairment and Neuropsychiatry Manifestation Following Mild and Moderate Traumatic Brain Injury at 3 Months and 6 Months

**DOI:** 10.21315/mjms2022.29.5.6

**Published:** 2022-10-28

**Authors:** Aizzat Othman, Zamzuri Idris, Azmin Kass Rosman, Jafri Malin Abdullah, Abdul Rahman Izaini GhanI, Ahmad Zabidin Zakaria

**Affiliations:** 1 Department of Neurosurgery, Hospital Sungai Buloh, Selangor, Malaysia; 2 Department of Neurosciences, School of Medical Sciences, Universiti Sains Malaysia, Kelantan, Malaysia; 3 Brain and Behaviour Cluster, School of Medical Sciences, Universiti Sains Malaysia, Kelantan, Malaysia; 4 Department of Psychiatry, Hospital Sungai Buloh, Selangor, Malaysia

**Keywords:** traumatic brain injury, mild, moderate, cognitive impairment, neuropsychiatry manifestation, Montreal cognitive assessment, General Health Questionnaire-12

## Abstract

**Background:**

Cognitive impairment (CI) and neuropsychiatry manifestation (NM) are known complications among patients with traumatic brain injury (TBI). However, the clinical correlation between mild and moderate TBI with the above have not been extensively studied.

**Methods:**

The patients (*n* = 54) were divided into mild and moderate TBI. Both groups were assessed at 3 months and 6 months post-trauma for the same measures. Diagnosis of CI was done using the Montreal cognitive assessment (MoCA) questionnaire while NM screening was performed using the 12-items General Health Questionnaire (GHQ-12) followed by MINI International Neuropsychiatry Interview (MINI).

**Results:**

We found five patients (19.2%) with mild TBI had CI and five patients (19.2%) had NM at 3 months. Only one patient (3.8%) persistently has CI at 6 months while the rest recovered. As for moderate TBI, 11 patients (39.3%) had CI and seven patients (25%) had NM at 3 months but none had persistent CI or NM at 6 months. Age (*P* < 0.05) and blood pressure were significant risks (*P* < 0.05) for CI and NM at 3 months.

**Conclusion:**

This study highlighted the importance of screening following mild and moderate TBI at 3 months and 6 months. Early recognition facilitates effective rehabilitation programmes planning hence improve prognosis in the future.

## Introduction

Cognitive impairment (CI) is a common consequence of traumatic brain injury (TBI). Impairment is particularly prominent in terms of information processing speed and attention, memory and executive functioning. Cognition is seen markedly impaired around 1-month post-injury (1). Similar observation was found by author that CI manifest between 48 h and 1 month after trauma. CI typically resolve in 80%–85% of cases of uncomplicated mild TBI within 3 months–6 months (2). Another study showed that most CI tend to disappear beyond 1-month post-trauma, but some do persist, thus suggesting different recovery patterns (3). Nonetheless, studies have also found that about 5%–10% of individuals experience the presence of persistent and atypical cognitive deficits up to 1 year (4). On the other hand, for moderate TBI survivors approximately 65% report to have long-term problems with cognitive functioning. These symptoms persist longer than mild TBI from several months up to several years after the trauma (5) and lead to a less favourable recovery (6). Factors that may influence cognitive outcome according to current literatures include demographic or premorbid characteristics, severity of injury, type and location of lesions, early cognitive functioning or event of post-traumatic amnesia (PTA) (7).

Similarly, neuropsychiatry manifestation (NM) is not uncommon after TBI. The term neuropsychiatry is a discipline that focuses on the relationship between the brain and its role in thinking, emotions and behaviour. Sequelae such as mood and anxiety disorders, post-concussive syndrome, personality change, aggression and psychosis are among the most common problems after TBI. Ciurli and colleagues (8) in their study reported the prevalence of psychiatric symptoms found in 120 persons with TBI are apathy (42%), irritability (37%), dysphoria/depressed mood (29%), disinhibition (28%), eating disturbances (27%) and agitation (24%). In one study of 939 TBI patients, the prevalence of any psychiatric illness in the first year was 49% following moderate to severe TBI and 34% following mild TBI (9). Another study found out that the prevalence of depression after TBI was approximately 30%. Based on their findings, on average 27% met criteria for depression 3 months–6 months from injury; 32% at 6 months–12 months; and 33% beyond 12 months (10). Anxiety disorders on the other hand frequently coexist with depressive disorders. Prevalence of anxiety disorders has been reported ranging from 18%–60% and generalised anxiety disorder (GAD) is the most commonly reported anxiety disorder after mild and moderate TBI ranging from 24%–27% (11). Risk factors for NM included female gender, younger age, lower education and preinjury unemployment (12). On top of that, other factors like increasing age, arteriosclerosis and alcoholism increased the risk of NM (13). Surprisingly, the effects of severity of TBI either mild or moderate TBI are uncertain. Studies using the Glasgow Coma Scale (GCS) score to estimate severity have generally not found an association between mild or moderate TBI to later psychiatric symptoms (14).

It is important to appreciate that TBIs not only damage brain structure but affect the brain function as well. Damage to the structures include skull fractures, extradural hematoma, subdural haematoma, brain contusion or diffuse axonal injury (15). These usually prompt urgent treatment via medical or surgical intervention. While damage to the function of the brain is rather insidious as it manifests gradually where NM and CI are such examples (16). Consequences following head trauma may lead to significant morbidity and mortality both in acute setting as well as long term sequelae. Therefore, it is imperative that clinicians be able to recognise and treat these conditions in order to more effectively manage head trauma, improve outcome and care for patients. Despite the emphasis placed upon physical deficits during the early stages of recovery from TBI, cognitive and behavioural deficits gave rise to the major morbidity which most impairs the capacity to return to work and maintain social activities (17). In this study, we wanted to determine whether CI and NM have any relation to severity of TBI especially in mild and moderate group. Understanding the demographics will have an impact on healthcare planning and the provision of resources and may help us meet the unique needs of these patients. Long-term outcomes of these patients are important for clinicians to assist patients and their families in formulating rehabilitation intervention and reducing secondary complications.

## Methods

This was a prospective cohort study which was conducted from October 2018 until October 2019. Study population was focused on TBI patients and subjects were recruited. Subjects were recruited from clinic during first follow-up visit at 6 weeks post-discharge followed by subsequent visit at 3 months and re-assessed again at 6 months visit.

**Figure 1:**
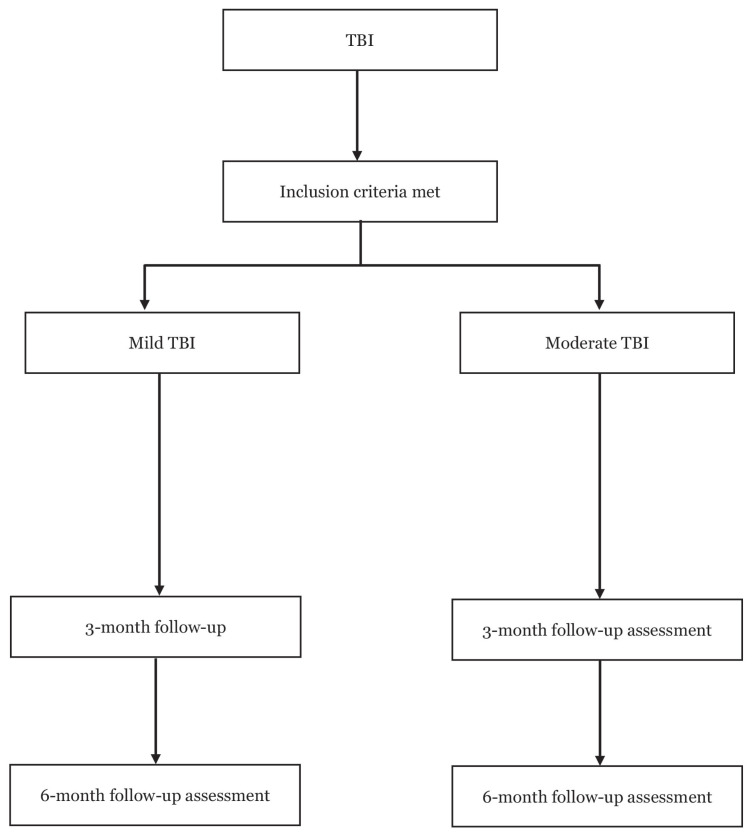
Study flowchart Note: TBI = traumatic brain injury

Fifty-four patients were recruited from the Neurosurgery Clinic in Hospital Kuala Lumpur, Malaysia. A purpose-designed questionnaire was used to collect information during interviews in the following areas: age, gender, race, educational status, civil status, employment status, smoking, alcohol, mechanism of trauma, GCS, CT brain findings and surgical procedure. First contact with subjects was during their immediate follow-up 6 weeks post-discharge from neurosurgical ward. Our inclusion criteria were: i) patient must be a TBI case; ii) patient’s GCS on admission was more than 8; iii) patient was above 18 years old and iv) patient must be able to complete the screening process. Any cases with GCS less than 8, penetrating brain injury, previous underlying psychiatry or cognitive impairment condition or any neurological disease, history of TBI before or not consented for the study will be excluded. Subjects were divided into mild and moderate group based on their admission GCS. Both mild and moderate TBI were assessed at 3 months and 6 months (Figure 1).

Cognitive status was assessed in both mild and moderate TBI patients using the Montreal cognitive assessment (MoCA) questionnaire. This set of questionnaire has been translated into Malay language and validated as mentioned in the literature review. MoCA questionnaire was used to assess neuropsychological functioning across its five domains (executive function, working memory, short-term memory, language and visuospatial ability). Executive function was assessed using trail-making, phonemic fluency and verbal abstraction tasks. Working memory was assessed using sustained attention, serial subtraction and digit span forward/backward tasks. Short-term memory was assessed through the delayed recall of five nouns. Language was assessed using naming (low familiarity animals), sentence repetition and the phonemic fluency task. Visuospatial ability was assessed using clock-drawing and cube-copying tasks. Any patient with score less than 26 will be regarded as having CI and further referred to neurorehabilitation unit for detail assessment and intervention.

**Figure 2:**
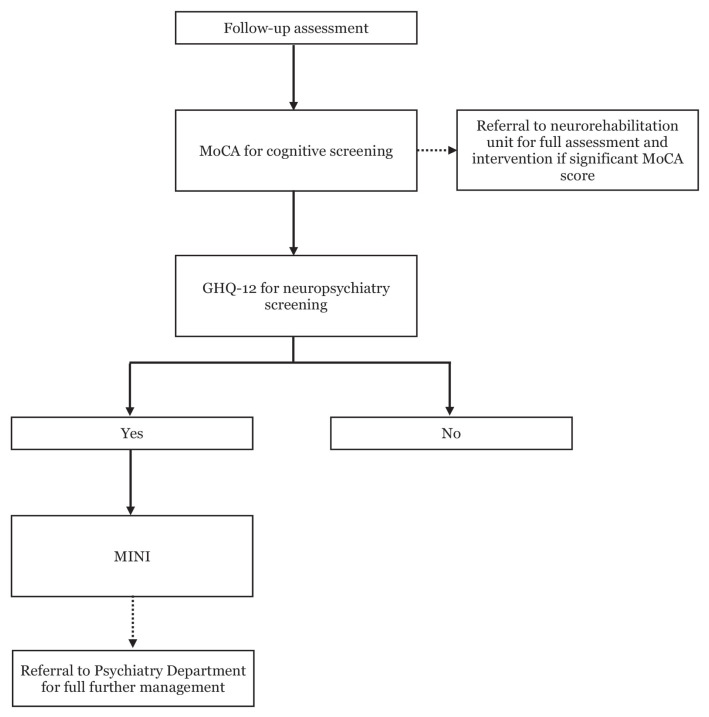
Flow during each assessment

Neuropsychiatry assessment was performed in two stages for both mild and moderate TBI patients. Firstly, all consented patients were screened with 12-items General Health Questionnaire (GHQ-12). Subjects who scored 4 or more on GHQ-12 will be followed by MINI International Neuropsychiatry Interview (MINI) which was compatible with international diagnostic criteria, including the tenth revision of the International Classification of Disease (ICD-10) as well as the Diagnostic and Statistical Manual of Mental Disorders (DSM). In this study, our focused was on neuropsychiatric diseases like major depression disorder (MDD), panic disorder and GAD. For any patient with scores to suggest any of the neuropsychiatry disease were then further referred to psychiatry clinic for detailed assessment and management (Figure 2).

The recorded data were analysed using the SPSS (version 22, SPSS Inc., IBM, Chicago, IL, USA) statistical software. Univariate analysis such as Chi-squared test used and logistic regression for multivariate analysis. Statistical significance was defined as *P* < 0.05 for all analyses, unless otherwise stated.

## Results

A total of 54 patients were recruited in which mild TBI were 26 patients (48.1%) and moderate TBI were 28 patients (51.9%). Their median age group were 27 years old (interquartile range 21–38) for mild TBI and 26 years old (interquartile range 23–34) for moderate TBI. Majority were male in both groups with male to female ratio of 3:1 for mild TBI and 2:1 for moderate TBI. Majority of the mechanism of injury were motor vehicle accident where highest for both groups involving motorcyclists; 76.9% for mild TBI and 71.4% for moderate TBI, respectively. Information pertaining to demographic details and injury-related characteristics of the patients for both mild and moderate TBI were presented in Table 1.

Subjects were divided into mild and moderate TBI and evaluated twice at interval of 3 months and 6 months. In mild TBI (*n* = 26), we found that five patients (19.2%) had CI and five patients (19.2%) had NM at 3 months. However, upon second assessment at 6 months, only one patient (3.8%) persistently had CI in mild TBI. As for moderate TBI (*n* = 28), 11 patients (39.3%) had CI and seven patients (25%) had NM at 3 months but none had it persistent at 6 months for both mild and moderate TBI.

Univariate analysis was carried out comparing the presence of CI and NM at 3 months and 6 months as dependent variables and severity of GCS as independent variables. Severity of GCS either mild or moderate TBI does not contribute significantly (*P* = 0.26) towards CI and NM at 3 months and 6 months (Table 2).

Multiple logistic regression analyses were carried out by using the presence of CI and NM at 3 months and 6 months as a dependent variables and the following risk factors as covariates: age, gender, employment status, income status, blood pressure on admission, CT brain findings either normal or abnormal. According to the logistic regression analyses, there was a statistically significant association between blood pressure and manifestation of CI at 3 months with *P* < 0.05.

There are statistically significant risk factors for CI in systolic blood pressure (OR: 1.223, Confidence interval: 1.001–1.495; *P* = 0.049) and diastolic blood pressure (OR: 1.215, Confidence interval: 1.016–1.454; *P* = 0.033) at 3 months. We found that an increase in a unit of systolic or diastolic blood pressure leads to the odds of the occurring of CI by 1.223 time for systolic and 1.215 for diastolic, respectively. As for NM at 3 months’ assessment, age was associated with increased risk of NM (OR: 0.678, Confidence interval: 0.463–0.995; *P* = 0.047). An increase in a unit of age leads to the odds of the occurring of NM by 0.678 time.

There were no statistically significant risk factors in both CI and NM at 3 months and 6 months. The results were tabulated in Table 3.

## Discussion

This study aimed at evaluating the presence of CI and NM following mild and moderate TBI at 3 months and 6 months. In addition, our study wanted to determine any association between risk factors with CI and NM.

The main result showed that mild and moderate TBI does not have any significant effect (*P* = 0.26) on CI and NM, hence, we were unable to reject our null hypothesis. Our findings are contradicting with similar study conducted where they evaluated 12 patients with mild and moderate TBI post-1-year injury and demonstrated that CI does correlate with severity of GCS (18).

These contradicting findings are attributed to the different sets of assessment tool used which may influence the detection rate. We chose MoCA questionnaire as our sole assessment tool because it’s a brief tool and require less than 10 min to complete the assessment hence its suitable for a busy outpatient neurosurgery clinic. It was reported to be a useful and psychometrically valid tool for the assessment of gross cognitive function in TBI patients (19). On another note, we used mostly (81.4%) translated Malay MoCA questionnaire for our assessment as Malay language is our national language. The original English version of the MoCA questionnaire was translated into Malay language and has been validated for cases like stroke (20) and even though characteristic for CI of post-stroke and TBI appear similar in certain aspects (21) as no study was done among TBI population using Malay MoCA questionnaire.

In pertaining to MoCA questionnaire as an assessment tool, studies reported that the significant of the scores were strongly dependent on educational level. They found that when applying the MoCA test on individuals, those with lower educational level seem to display higher rates of CI. It was suggested in the study that different cut-off values were required in order to better distinguish normality from CI in the lower educational level group (22). In this study, 92.3% of mild TBI and 82.1% of moderate TBI received secondary to tertiary education hence made the assessment valid in this population.

However, the trend of our data appeared similar with other studies. We found that the presence of CI at 3 months among mild and moderate TBI patients were 19.2%–39.3%. This finding was consistent to what was reported by de Boussard et al. (23) where they found that 30% of their patients persisted to have CI at 3 months following mild TBI. Skandsen et al. (24) found that 43% of moderate TBI had CI at 3 months post-injury. Furthermore, at 6 months following mild and moderate TBI, nearly all patients (75%) recover except one patient persist to have CI. Similar findings were reported where one third of mild TBI patients continue to experience CI post-3-months injury and nearly 80%–85% of them fully recovered within 6 months (25).

As for NM, Jorge et al. (26) assessed 91 consecutive TBI patients (44% mild, 32.5% moderate and 23.2% severe) admitted to general hospital at 3 months, 6 months, 9 months and 12 months post-injury. Evaluation was done by psychiatrist and they found that in the TBI sample, 46.7% in mild TBI and 40% in moderate TBI had depression. Other study by Levin et al. (27) looked into depression at 3 months post-mild and -moderate TBI found out that 18% of mild TBI and 11% of moderate TBI had depression. Their patients were evaluated using structured psychiatric interviews and DSM criteria for diagnosis. In this study, we found that 19% of mild TBI and 39.3% of moderate TBI had depression which is similar range of percentage with both studies. Unfortunately, we couldn’t get any significant association between severity GCS and NM. We postulate that it may be affected by the fact that our interview session was set in a less conducive setup because of high number of patients in neurosurgical clinic. Therefore, the result may appear under reported.

In this study, we found that systolic and diastolic blood pressure correlate significantly with CI at 3 months’ post-injury regardless of either mild or moderate TBI. There’s no recent study to highlight the association of elevated blood pressure in TBI patients with CI. Szarka and colleagues (28) in their animal study managed to show the significance of this factor pertaining to CI in TBI rodents. They found out in the TBI sample with hypertension showed impaired learning and memory two weeks after mild TBI. Both condition eventually leads to persistent disruption of the blood-brain barrier, which was associated with accumulation of toxic blood borne substances in the brain parenchyma, neuroinflammation and cognitive decline amongst the animals.

We also found that the risk of having NM at 3 months post-injury is higher with increasing age. Similar finding was reported with previous literature by Glenn (29). Glenn found that NM particularly depression increases with age after injury. Based on their cohort, 41 patients (56% mild, 17% moderate and 27% severe TBI) with the mean age of 43.6 years old was evaluated using Beck Depression Investory-II (BDI-II) by three clinician investigators showed significant correlation between depression and increasing age. Another study by Levin et al. (30) showed similar significant association between increasing age and depression. Out of 41 patients with mild and moderate TBI, 31% were depressed at 1 month with mean age of 40 years old. Both studies share the same range of age for their cohort but compared to our study, our median age for mild and moderate TBI are ranging between 26 years old and 27 years old, hence it’s difficult to conclude despite its statistically significant. This does not entirely sure to indicate age is associated with depression, but rather suggest that it would take a larger sample size to determine this with greater certainty.

To our knowledge, this is the first study in Malaysia looking into association of TBI with CI and NM. Despite other reason stated above, another limitation of this study including limited sample size and inter interviewer factor as all interviews were conducted by a single researcher. In the future, we suggest recruiting larger sample size and to use additional assessment tool besides MoCA questionnaire alone. Multiple researchers especially during assessment should be assembled to prevent any bias to the outcome.

## Conclusion

TBI is common among young adults in Malaysia and little attention has been paid to the significant morbidity which may interfere with rehabilitation due to CI and NM as a consequence of TBI. While this study does not offer a conclusive answer pertaining to the association of mild and moderate TBI with CI and NM but it does provide some findings to enlighten treating clinicians. Regardless, future research should aim to replicate results in a larger sample with multiple assessment tool.

## Figures and Tables

**Table 1:** Demographic details and injury-related characteristics with mild and moderate TBI

Variable and subcategory	Subjects with TBI (*n* = 54)		*P*-value

	Mild (*n* = 26)	Moderate (*n* = 28)	
Age; median (IQR)	27 (20–42)	26 (23–34)	0.90
Gender			0.17
Male	21 (80.7)	18 (64.3)	
Female	5 (19.3)	10 (35.7)	
Education level			0.16
No education	0 (0.0)	2 (7.1)	
Primary	2 (7.7)	3 (10.7)	
Secondary	22 (84.6)	14 (50.0)	
Tertiary	2 (7.7)	9 (32.1)	
Civil status			0.51
Married	15 (57.7)	15 (53.6)	
Single	10 (38.5)	13 (46.4)	
Widowed	1 (3.8)	0 (0.0)	
Income			0.59
Low	25 (96.2)	26 (92.9)	
Middle	1 (3.8)	2 (7.1)	
Employment status			0.52
Employed	17 (65.4)	21 (75.0)	
Unemployed	1 (3.8)	5 (17.9)	
Student	4 (15.4)	0 (0.0)	
Retired	4 (15.4)	2 (7.1)	
Smoking status			0.29
Yes	12 (46.2)	9 (32.1)	
No	14 (53.8)	19 (67.9)	
Alcohol intake			0.09
Yes	0 (0.0)	4 (14.3)	
No	26 (100)	24 (85.7)	
Mechanism of trauma			0.54
MB	20 (76.9)	20 (71.4)	
Car	0 (0.0)	4 (14.3)	
Pedestrian	1 (3.8)	0 (0.0)	
Others	5 (19.2)	4 (14.3)	
Blood pressure on arrival			
Systolic; mean (SD)	125 (9.4)	125 (7.5)	0.35
Diastolic; mean (SD)	78 (8.2)	78 (8.2)	0.29

Notes: Values are number of cases (%) unless otherwise stated; IQR = interquartile range

**Table 2:** CI and NM at 3 months and 6 months for mild and moderate TBI

		CI		NM	

		Yes	No	Yes	No
3 months GCS	Mild (*n* = 26)	5 (19.2)	21 (80.7)	5 (19.2)	21 (80.7)
	Moderate (*n* = 28)	11 (39.3)	17 (60.7)	7 (25.0)	21 (75.0)
6 months GCS	Mild (*n* = 26)	1 (3.8)	25 (96.2)	0	26 (100.0)
	Moderate (*n* = 28)	0	28 (100.0)	0	28 (100.0)

Notes: Values are number of cases (%) unless otherwise stated

**Table 3:** Association between risk factors with CI and NM at 3 and 6 months in both mild and moderate TBI

	3 months		6 months	

	CI	NM	CI	NM
Age	0.24	0.047	0.992	0.621
Gender				
Male versus Female	0.076	0.202	0.997	0.998
Alcohol				
Yes versus No	0.557	0.249	0.618	0.732
Educational status				
Low versus High	0.566	0.251	0.546	0.621
Employment status				
Employed versus Unemployed	0.658	0.117	0.995	0.914
Income status				
Low versus Middle	1.000	1.000	0.999	0.997
Blood pressure				
Systolic	0.049	0.2	0.991	0.463
Diastolic	0.033	0.044	0.994	0.488
CT brain				
Normal versus Abnormal	0.280	0.094	0.998	0.997

Notes: Values are *P*-value
